# Anticoagulation Therapy After Biologic Aortic Valve Replacement

**DOI:** 10.3389/fcvm.2021.698784

**Published:** 2021-06-21

**Authors:** Monna E. Myllykangas, Tuomas O. Kiviniemi, Jarmo M. Gunn, Veikko V. Salomaa, Arto Pietilä, Teemu J. Niiranen, Jenni Aittokallio

**Affiliations:** ^1^Department of Anesthesiology, Intensive Care, Emergency Care, and Pain Medicine, University of Turku, Turku, Finland; ^2^Division of Perioperative Services, Intensive Care Medicine, and Pain Management, Turku University Hospital, Turku, Finland; ^3^Heart Centre, Turku University Hospital, Turku, Finland; ^4^Department of Internal Medicine, University of Turku, Turku, Finland; ^5^Division of Surgery, University of Turku, Turku, Finland; ^6^Department of Public Health and Welfare, Finnish Institute for Health and Welfare, Helsinki, Finland; ^7^Division of Medicine, Turku University Hospital, Turku, Finland

**Keywords:** aortic valve, heart valve surgery, anticoagulation, bioprosthetic valve, heart valve disease

## Abstract

**Objectives:** Thromboembolism prophylaxis after biologic aortic valve replacement (BAVR) is recommended for 3 months postoperatively. We examined the continuation of oral anticoagulation (OAC) treatment and its effect on the long-term prognosis after BAVR.

**Methods:** We used nation-wide register data from 4,079 individuals who underwent BAVR. We examined the association between warfarin and the non-vitamin K antagonist oral anticoagulant use with death, stroke and major bleeding in 2010 – 2016.

**Results:** The risk of stroke was higher (HR 2.39, 95% CI 1.62 – 3.53, *p* < 0.001) and the risk of death was lower (HR 0.79, 95% CI 0.65 – 0.96, *p* = 0.016) in OAC-users compared to individuals without OAC. We observed no significant associations between OAC use and bleeding risk.

**Conclusion:** OAC use after BAVR was associated with increased risk of stroke and decreased risk of death. These observational findings warrant validation in randomized controlled trials before any clinical conclusions can be drawn.

## Introduction

Thromboembolism prophylaxis after biologic aortic valve replacement (BAVR) is recommended for the first 3 months after operation either with warfarin or low-dose aspirin (75–100 mg daily) (1–3). The peak incidence of thromboembolic complications occurs during this period, most likely reflecting the lack of endothelialization of the newly implanted materials (4) as the risk markedly decreases already 90 days after the operation (1). Yet, oral anticoagulation therapy (OAC) often continues in many patients after the first 3-month period due to atrial fibrillation (AF) or other conditions that increase the risk of thromboembolic complications.

Warfarin is currently the only OAC with an indication for early thromboembolism prophylaxis after BAVR (2, 3). However, warfarin has limitations related to its complicated dosing. Therefore, non-vitamin K antagonist oral anticoagulants (NOACs) are increasingly used as they are known to prevent stroke or thromboembolisms at least as well as warfarin in AF patients (5, 6). NOACs may also be used in patients with AF and valvular heart disease (VHD) excluding moderate-to-severe mitral stenosis and mechanical heart valves (2, 5, 6).

In the present study, we examined the prevalence and type of OAC treatment after the mandatory 3-month warfarin treatment period in patients who have undergone BAVR operation. We also assess the association of OACs with complications and death.

## Methods

### Patient Population

We used data of the Finnish Cardiovascular Diseases Register that was formed by combining individual patient data from three nationwide electronic health care registers: The National Hospital Discharge Register, the National Drug Reimbursement Register and the Causes of Death Register. The information on diagnoses and procedures was collected from the National Hospital Discharge Register. The recording of the diagnoses to the registers is obligatory in Finland and done by the treating physicians using codes from the International Classification of Diseases 10th revision (ICD-10). The study period was from 2010 to 2016. The institutional ethics review board of the National Public Health Institute has approved the research use of the Finnish Cardiovascular Disease Register.

The data on drug purchases for reimbursed medications was gathered from the Drug Reimbursement Register 3 months after the operation (after obligatory warfarin period). The patients were grouped according to the medication use. If the medication was discontinued or changed during the study period, the patient was changed into another group after 3 months from the latest drug purchase.

The surveyed drugs were warfarin and the NOACs (rivaroxaban, edoxaban, dabigatran, and apixaban). The drug purchases were surveyed in 3 month-periods to detect a change of the drug or discontinuation of medication. In addition, the information on the causes of death was added from the National Causes of Death Register. For the present study, we used data over 7 years from the January 1, 2010 to December 31, 2016 and the median of the follow up time was 3.0 years.

### Endpoints

Three primary endpoints were used: death, stroke and incidence of major bleeding (gastrointestinal bleeding or intracranial non-traumatic bleeding, excluding subarachnoid hemorrhage as it is mainly aneurysm-based). Intracranial bleeding was defined with ICD-10 codes I61 (intracerebral hemorrhage) and I62 (subdural hemorrhage). The gastrointestinal bleeding was defined with ICD-10 codes I85 (esophageal varix bleeding), K22.6 (Mallory-Weiss syndrome), K25-29 (ulcers), K62.5 (hemorrhage of anus and rectum), K66.1 (hemoperitoneum) and K92 (hematemesis and melena). Stroke was defined with the ICD-10 codes I63 (ischemic stroke) and I64 (stroke, not specified).

### Statistics

The risk of adverse outcomes was assessed using Cox proportional hazards regression models adjusted for age, sex, procedure urgency, previous myocardial infarction, previous stroke, concomitant coronary artery bypass, diabetes, hypertension, chronic lung disease and previous atrial fibrillation. Anticoagulation status with three possible states (no-OAC, warfarin, or NOAC) was included in the model as the state that was current at the time of the outcome event. Medications are dispensed in 3-month quantities in Finland. If the medication was discontinued or changed, the patient was changed into another group after 3 months from the latest drug purchase. Therefore, an individual patient can be included both in the warfarin- and NOAC-groups if both medications were purchased during the 3 months preceding end of follow-up. Baseline for the Cox models was determined as three months after the operation. All statistical analyses were carried out using R statistical software version 3.6.0 (R Foundation for Statistical Computing, Vienna, Austria).

## Results

Between 2010 and 2016, altogether 4,079 patients had first-ever open-heart BAVR. During the first 3 post-operative months, 199 (4.9 %) of the patients died, 28 (0.7%) had non-traumatic intracranial or gastrointestinal bleeding and 108 (2.6%) had a stroke.

The characteristics of BAVR patients 3 months after the operation are presented in Table 1. In total, 3,880 patients (95.1%) were alive 3 months after the operation. Of those, 2,245 were on OAC and 1,635 were not (no-OAC). Warfarin use was more common than NOAC use as only 7.5% of the patients used NOAC during the follow-up. The share of women was equal in all groups (42.6 – 44.6%). However, the patient groups differed in terms of age and concomitant diseases. OAC-patients were older, had more often diabetes, hypertension, chronic obstructive lung disease and previous AF. In addition, warfarin patients had more often a prior myocardial infarction or stroke, and their operations were more often urgent (Table 1).

**Table 1:** Characteristics of BAVR patients 3 months after the operation between 2010 and 2016 (*n* = 3,880).

**Characteristic**	**Any OAC**	**Warfarin**	**NOAC**	**No-OAC**
*N*	2,245	2,158	168	1,635
Age, y	75.4 (7.1)	75.5 (7.1)	74.9 (5.9)	72.6 (8.8)
Women	958 (42.7)	920 (42.6)	75 (44.6)	715 (43.7)
Urgent	378 (16.8)	370 (17.1)	23 (13.7)	225 (13.8)
Previous MI	271 (12.1)	264 (12.2)	19 (11.3)	164 (10.0)
Previous stroke	215 (9.6)	209 (9.7)	12 (7.1)	99 (6.1)
Concomitant CABG	246 (11.0)	236 (10.9)	19 (11.3)	206 (12.6)
Diabetes (type 1 or 2)	707 (31.5)	679 (31.5)	60 (35.7)	432 (26.4)
Hypertension	1370 (61.0)	1311 (60.8)	103 (61.3)	821 (50.2)
Chronic lung disease	353 (15.7)	340 (15.8)	32 (19.0)	197 (12.0)
Previous AF	748 (33.3)	728 (33.7)	45 (26.8)	76 (4.6)

*Patients were grouped according to the medication use at the end of follow-up. If the medication was discontinued or changed, the patient was changed into another group after 3 months from the latest drug purchase. Therefore, an individual patient can be included both in the warfarin- and NOAC-groups if both medications were purchased during the 3 months preceding end of follow-up. A procedure was defined as urgent if it was necessary to perform within 1 week.*

*Numbers are mean (SD) for age and n (%) for other variables. p-values any OAC vs. no-OAC, Warfarin vs. no-OAC and NOAC vs. no-OAC*.

*AF, atrial fibrillation; MI, myocardial infarction; BAVR, biologic aortic valve replacement; CABG, coronary artery bypass grafting; OAC, oral anticoagulation; NOAC, non-vitamin K antagonist oral anticoagulant*.

The incidence rates of complications grouped by medication use for the time period 3 months after the operation till the end of the follow-up are presented in Table 2. There was no difference in bleeding complications between OAC and no-OAC groups. However, strokes were more common in patients with OAC, 17.3/1,000 person-years and 19.7/1,000 person-years in patients with warfarin and NOAC, respectively. In the NOAC group, the incidence of death was markedly lower than in the no-OAC group (*p* = 0.0002).

**Table 2:** Bleeding complications and deaths for the time period 3 months after the operation until the end of the follow-up (*n* = 3880).

**Incidence after 3 months post-operatively**	**Warfarin *n* = 2,158**	***p*-value**	**NOAC *n* = 168**	***p*-value**	**no-OAC *n* = 1,635**
Median follow-up, years	3.2		3.1		2.9
Bleeding complication, *n* (*n*/1,000 person years)	48 (6.8)	0.559	4 (7.2)	0.744	29 (5.9)
Stroke, *n* (*n*/1,000 person years)	122 (17.3)	<0.0001	11 (19.7)	0.039	35 (7.2)
Death, *n* (*n*/1,000 person years)	342 (48.5)	0.089	10 (17.9)	0.0002	204 (41.9)

*Patients are grouped according to the medication use at the end of follow-up. If the medication was discontinued or changed, the patient was changed into another group after 3 months from the latest drug purchase. Therefore, an individual patient can be included both in the warfarin- and NOAC-groups if both medications were purchased during the 3 months preceding end of follow-up. Bleeding complication = Intracranial non-traumatic bleeding or gastrointestinal bleeding. p-values Warfarin vs. no-OAC and NOAC vs. no-OAC. OAC, oral anticoagulation; NOAC, non-vitamin K antagonist oral anticoagulant*.

The risk of death was significantly lower in patients using OAC (Table 3). The lower risk of death was seen despite the increased risk of stroke in the OAC-group (Table 3).

**Table 3:** Risk of complications in OAC users vs. no-OAC patients.

	**Warfarin vs. no-OAC**		**Any OAC vs. no-OAC**	
**Complication**	**HR (95% CI)**	***p*-value**	**HR (95% CI)**	***p*-value**
Any complication	1.03 (0.87–1.23)	0.700	1.01 (0.85–1.20)	0.913
Bleeding complication	1.00 (0.61–1.65)	0.996	1.00 (0.61–1.65)	0.995
Stroke	2.45 (1.66–3.21)	<0.001	2.39 (1.62–3.53)	<0.001
Death	0.82 (0.67–0.99)	0.039	0.79 (0.65–0.96)	0.016

*Patients are grouped according to the medication use at the end of follow-up (warfarin only or all the OACs including warfarin = any OAC). If the medication was discontinued or changed, the patient was changed into another group after 3 months from the latest drug purchase. Hazard ratios are warfarin vs. no-OAC and any OAC vs. no-OAC. The models are adjusted for age, sex, procedure urgency, previous myocardial infarction, previous stroke, concomitant CABG, diabetes, hypertension, chronic lung disease and previous atrial fibrillation. CI, Confidence interval; HR, Hazard ratio; OAC, oral anticoagulation; CABG, coronary artery bypass grafting*.

## Discussion

Over the 7-year study period, 4,079 patients had their first-ever BAVR in Finland. Of these patients, 3,880 were alive 3 months post-operatively. We studied the use of OACs after the first 3 months post-operatively. There was no difference in bleeding complications between the OAC-group and no-OAC group, but the incidence of stroke was higher in OAC users. However, the risk of death was markedly lower in the OAC group than in the no-OAC group.

As expected, stroke incidence was higher in OAC users than in non-users most likely due to underlying higher stroke risk, comorbidity burden and higher AF incidence (Table 1). It is well-known that patients in AF as compared to those in sinus rhythm are at 20 – 25% higher stroke-risk, and this risk further increases when concomitant risk factors are present (7). It is noteworthy, however, that despite OAC treatment, the risk of stroke remained high. This may also be due to suboptimal therapeutic use of warfarin for fear of overdose.

Patients with AF and VHD have higher risk of bleeding complications than patients with AF solely (8). In AF patients, NOAC use results in reduced bleeding risk compared to warfarin (9). In patients with AF and VHD, NOACs (excluding rivaroxaban) also reduce the risk of bleeding complications (8). However, in the current study, we observed no difference in bleeding complications between the OAC and no-OAC groups. Cardiac surgery patients are followed often and regularly, and are therefore possibly more likely to avoid hazardous warfarin overdosing than other AF patients.

We also observed that the risk of death was slightly, but significantly lower in patients using OACs than in non-users (Table 3). However, the anticoagulants increase the risk of bleeding, so there is no evidence to support their long-term use unless they have an indication other than the biologic valve (2). The higher mortality risk in patients without OACs could be a result of medications being stopped in patients with a poor prognosis or the patient being in long-term inpatient care without pharmacy drug purchases that are stored in the Drug Reimbursement Register.

Our results are limited by their observational nature and they should be viewed as hypothesis-generating and provide interesting information on the use and effects of OACs. Use of any OAC (NOAC or warfarin) was related to reduced risk of death, but the small number of NOAC users prevented us from analyzing NOAC and warfarin users separately in the multivariable models. Because our results are based on register data, we only had information on drug purchases, not whether the drugs were actually taken. Moreover, we do not have precise information on the reasons why the medications were stopped or initiated. Also, our data only provides information on whether the procedure was performed with a biologic valve and no information on specific valve types or surgical techniques. We neither had the information on renal insufficiency or dialysis during the study period. In addition, ICD codes for valve thrombosis were not available, rendering it was impossible to determine whether valve thrombosis occurred after discontinuation of the OACs. Our study does not also include transcatheter aortic valve replacement procedures, as data on these procedures have only been available in our register from 2016.

In high risk patients, the use of OACs appears to be associated with reduced risk of death and increased risk of stroke. Despite OAC use, the incidence of bleeding complications was similar to that in no-OAC patients. These observational findings warrant validation in randomized controlled trials before any clinical conclusions can be drawn.

## Data Availability Statement

The datasets presented in this study are not publicly available due to their sensitive nature. Further inquiries can be directed to the corresponding author/s.

## Author Contributions

All authors have contributed to the design, content, drafting of the manuscript, read, and approved the submission of the manuscript.

## Conflict of Interest

The authors declare that the research was conducted in the absence of any commercial or financial relationships that could be construed as a potential conflict of interest.
